# Perturbed body fluid distribution and osmoregulation in response to high salt intake in patients with hereditary multiple exostoses

**DOI:** 10.1016/j.ymgmr.2021.100797

**Published:** 2021-11-05

**Authors:** Jetta J. Oppelaar, Nienke M.G. Rorije, Rik H.G. Olde Engberink, Youssef Chahid, Naomi van Vlies, Hein J. Verberne, Bert-Jan H. van den Born, Liffert Vogt

**Affiliations:** a Amsterdam UMC, University of Amsterdam, Department of Internal Medicine, Section of Nephrology, Amsterdam Cardiovascular Sciences, Meibergdreef 9, Amsterdam, the Netherlands; b Amsterdam UMC, University of Amsterdam, Department of Radiology and Nuclear Medicine, Meibergdreef 9, Amsterdam, the Netherlands; c Amsterdam UMC, University of Amsterdam, Laboratory Genetic Metabolic Diseases, Meibergdreef 9, Amsterdam, the Netherlands; d Amsterdam UMC, University of Amsterdam, Department of Internal Medicine, Section of Vascular Medicine, Amsterdam Cardiovascular Sciences, Meibergdreef 9, Amsterdam, the Netherlands

**Keywords:** Sodium, Glycosaminoglycans, Heparan sulfate, Hereditary Multiple Exostoses, Water balance, Osmoregulation, BMI, Body mass index, BP, Blood pressure, ECFV, Extracellular fluid volume, EXT1/EXT2, Extosin-1 / Extosin-2, GAG, Glycosaminoglycan, HME, Hereditary Multiple Exostoses, HSD, High sodium diet, ICFV, Intracellular fluid volume, IFV, Interstital fluid volume, LSD, Low sodium diet, PV, Plasma volume, TBW, Total body water

## Abstract

**Background:**

Hereditary Multiple Exostoses (HME) is a rare autosomal disorder characterized by the presence of multiple exostoses (osteochondromas) caused by a heterozygous loss of function mutation in *EXT1* or *EXT2*; genes involved in heparan sulfate (HS) chain elongation. Considering that HS and other glycosaminoglycans play an important role in sodium and water homeostasis, we hypothesized that HME patients have perturbed whole body volume regulation and osmolality in response to high sodium conditions.

**Methods:**

We performed a randomized cross-over study in 7 male HME patients and 12 healthy controls, matched for age, BMI, blood pressure and renal function. All subjects followed both an 8-day low sodium diet (LSD, <50 mmol/d) and high sodium diet (HSD, >200 mmol/d) in randomized order. After each diet, blood and urine samples were collected. Body fluid compartment measurements were performed by using the distribution curve of iohexol and ^125^I-albumin.

**Results:**

In HME patients, HSD resulted in significant increase of intracellular fluid volume (ICFV) (1.2 L, *p* = 0.01). In this group, solute-mediated water clearance was significantly lower after HSD, and no changes in interstitial fluid volume (IFV), plasma sodium, and effective osmolality were observed. In healthy controls, HSD did not influence ICFV, but expanded IFV (1.8 L, *p* = 0.058) and increased plasma sodium and effective osmolality.

**Conclusion:**

HME patients show altered body fluid distribution and osmoregulation after HSD compared to controls. Our results might indicate reduced interstitial sodium accumulation capacity in HME, leading to ICFV increase. Therefore, this study provides additional support that HS is crucial for maintaining constancy of the internal environment.

## 1 Introduction

Hereditary Multiple Exostoses (HME), also known as Hereditary Multiple Osteochondromas, is a rare genetic disorder with a reported prevalence between 1:50000 to 1:100000 affected individuals in the general population [[1], [2]]. HME is characterized by growth of multiple begin bone tumors (exostoses or osteochondromas) [3]. The autosomal dominant inheritance pattern of HME has been linked to mutations in *EXT1* (OMIM: 608177) and *EXT2* (OMIM: 608210), both coding for enzymes involved in heparan sulfate (HS) polymerization [[4], [5], [6], [7]]. HS is a member of the glycosaminoglycan (GAG) family, consisting of negatively charged linear polysaccharides, which are abundantly expressed in various tissues. Chains of HS are covalently linked to a core protein, resulting in combined molecules known as HS proteoglycans, which represent the major component of extracellular matrix in humans [[8], [9]]. Studies into HS structure in patients with HME have shown that their loss of function mutations in *EXT1* or *EXT2* led to altered tissue and plasma HS composition [[10], [11], [12]]. Besides the influence of HS on important processes in skeletal growth and morphogenesis, studies into sodium balance also revealed an important role for sulfated GAGs, such as HS, in sodium and water homeostasis [[13], [14], [15]].

Sodium is the major cation in the extracellular fluid compartment and is traditionally seen as regulator of extracellular fluid volume (ECFV). This prevailing assumption states that during increased sodium intake, consistency of the internal environment is reached by both increased water intake and increased renal water reabsorption. Consequently, this will result in augmentation of ECFV and body weight, eventually leading to a subsequent rise in blood pressure (BP). However, carefully conducted, long-term sodium studies in humans have revised our understanding of sodium homeostasis and its close link to ECFV [[16], [17], [18], [19]]. These studies revealed that GAG-rich tissues facilitate sodium accumulation in concentrations far exceeding those in plasma [[20], [21], [22]]. Experimental studies have shown that a high tissue sodium content is accompanied by both increased GAG synthesis as well as GAG chain elongation [13]. For example in human arteries and muscle higher xylosyltransferase-1 (XYLT-1, enzyme that initiates GAG synthesis) expression coincides with higher tissue sodium content [14].

In heterozygous *EXT1* and *EXT2* knock-out mice, it was shown that this genetic mutation significantly reduced sodium storage capacity, which coincided with volume depletion in these animals [15]. We questioned whether HME patients, characterized by changed tissue and plasma HS composition due to heterozygous *EXT1* or *EXT2* mutations, would respond differently to a high sodium diet with regard to the regulation of sodium, and osmotic and water balance. With this study we aimed to determine how HME patients respond to high and low dietary sodium intake, and if intact HS polymerization is critical for maintaining stability of the milieu interieur. We therefore assessed body fluid distribution and osmoregulation of male HME patients in response to high and low sodium intake as compared to age-matched healthy controls.

## 2 Methods

### 2.1 Participants and ethics approval

We performed two similar randomized cross-over intervention studies in young HME patients and healthy controls, respectively (between March 2013 and July 2015). Male, non-smoking individuals between 18 and 40 years old, with BP below 140/90 mmHg and body mass index (BMI) <30 kg/m^2^ were included. All participants had to have a stable and normal renal function (defined as no proteinuria and creatinine clearance >60 mL/min). HME patients had to have a documented medical diagnosis of HME, based on the combination of two or more documented osteochondromas, and on a suggestive familial autosomal dominant inheritance pattern [1]. HME patients were recruited by their outpatient orthopedic surgeon or by advertisements of the MHE-MO association of the Netherlands. Subjects with a history of renal or cardiovascular disease were excluded. Both studies were conducted in the Amsterdam UMC, location AMC, the Netherlands, after approval of the local ethics committee. All subjects provided written informed consent and the studies were conducted in accordance with the Declaration of Helsinki.

### 2.2 Dietary sodium intervention

All study subjects followed an 8-day low sodium diet (LSD, <50 mmol sodium/day) and high sodium diet (HSD, >200 mmol sodium/day) in a randomized order. Between diets there was a wash-out period of 1–2 weeks, in which study subjects adhered to their normal diets. The diet order was determined by block randomization via sealed envelopes by the study investigators. During follow-up, diets were not masked for the study subjects nor investigators. Diets were pursued with the help of a dietary list containing high and low sodium food products, on which study subjects could compile their own diet. Dietary compliance was checked by measuring 24-h sodium excretion on day 3, 6, and 8.

### 2.3 Laboratory and hemodynamic measurements

On day 8 of each diet, the study subject visited our research department after an overnight fast for blood sampling, assessment of osmoregulation and osmotic balance, and hemodynamic measurements. The measurements of the 24-h urine osmolality, total urine volume and plasma osmolality on day 8 were used to calculate solute-mediated water clearance, which indicates to what extent the diuresis is driven by urinary solutes. For the electrolyte-free water clearance, indicating to what extent urine is diluted, 24-h urine sodium and potassium concentration, total urine volume and plasma sodium were used [23]. Effective plasma osmolality was calculated as the sum of plasma concentrations of sodium, bicarbonate, chloride and glucose. We measured brachial BP in a supine position with a validated semi-automatic device (Omron 705 IT, OMRON Healthcare, the Netherlands). After at least 10 min of supine rest, we performed five sequential measurements of which the mean of the last two readings was used for analysis. To assess differences in body GAG composition, 24-h urinary GAG excretion was also measured on day 8 of LSD to quantify urinary excretion of HS disaccharides. In short, with GAG-specific enzymes GAGs were enzymatically digested into disaccharides, and the results were reported as previously described [[24], [25]]. A validated high performance liquid chromatography with mass spectrometry/mass spectrometry, capable of precisely distinguish HS disaccharides from other GAGs, was used to quantify urinary excretion of HS. Values below the lower limit of quantification were assigned values of half the lower limit of quantification. The disaccharide concentrations were adjusted for creatinine concentrations of the 24-h urine samples. The results of 24-urinary GAG excretion have been described in a previous publication [[26], [27]].

### 2.4 Measurement of body fluid compartments

On day 8 of both diets, two intravenous catheters were placed in the left and right antecubital veins. Fluid intake during these measurements was standardized to 875 mL for all participants. ECFV was measured by using iohexol (Omnipaque® 647 mg iohexol/mL), a nonionic radiopaque water soluble contrast agent, according to the method of Zdolsek et al. [28] Each subject was administered with 10 mL of iohexol through one of the intravenous catheters. After infusion we drew blood samples at regular time intervals (*t* = 0, 5, 10, 15, 20, 60, 90, 120, 150, 180 and 240 min after infusion). To calculate ECFV, we fitted a two-compartment kinetic model with an expected distribution phase of approximately 20 min to the data [28]. To account for the water content of plasma, we multiplied the obtained values by 0.934 [29].

Radiolabeled human serum albumin (^125^I-albumin) was used to measure plasma volume. We intravenously administered ^125^I-albumin solution of 100 kBq in 5 mL saline. From the intravenous catheter on the contralateral arm, we drew blood samples at regular time intervals (*t* = 0, 5, 10, 15, 20, 30, 45, 60 min after infusion). At 60 min after infusion we obtained one urine sample. In blood and urine samples we measured radioactivity, using a scintillation detector (Wizard^2^ 2480 Automatic Gamma Counter (PerkinElmer, USA)) measuring in duplicate with a coefficient of variation of <3%. Routine quality controls of the gamma counter were performed, according to the standard GLP features of PerkinElmer. For the determination of plasma volume we calculated the y-intercept of the disappearance curve of ^125^I-albumin in plasma, corrected for the injected dose of the tracer, according to the method described by Kreel et al. [30] PKSolver, a free Microsoft Excel add-in validated for pharmacokinetic and pharmacodynamic data analysis was used for above described ECFV and plasma volume calculations [31].

We calculated interstitial fluid volume (IFV) by subtracting the plasma volume from the ECFV. Also, ICFV was measured indirectly as the differences between total body water (TBW) and measured ECFV. By taking 60% of body weight, which was measured at day eight of each diet, we calculated TBW.

### 2.5 Statistics

Continuous data are shown as mean and standard deviation (SD) for data following a normal distribution and median with interquartile range (IQR) for non-parametric variables. Differences between LSD and HSD were assessed by using pared *t*-test or Wilcoxon rank sum test, depending on the data distribution. To investigate differences between HME patients and healthy controls, an unpaired t-test or Kolmogorov-Smirnov test was used as appropriate. Spearman's correlation coefficient was used to test correlations between variables. All statistical analyses were performed using IBM SPSS Statistics (version 26.0, IBM, USA). Figures were acquired using GraphPad prism (version 8 for Windows, GraphPad Software, USA). A *p*-value of ≤0.05 was considered significant.

## 3 Results

### 3.1 Study population and dietary intervention

We screened 8 HME patients and 19 healthy controls. Of the HME patients, one subject withdrew informed consent after screening due to inability to adhere to the protocol instructions. Before randomization, four healthy subjects withdrew their informed consent and three were excluded as a result of high BP (1 subject) or to blood drawing difficulties (2 subjects). Therefore, we included in our study 7 HME patients and 12 healthy controls with a mean age of, respectively, 26 (8) and 22 (40) years, normal kidney function and body mass index. Five HME patients had a confirmed mutation in *EXT1* or *EXT2* and two others were diagnosed based on their typical radiological HME phenotype. Baseline characteristics determined at screening (before start of the diets) are depicted in Supplementary Table 1. There was no loss-to-follow-up and all randomized subjects adequately followed dietary instructions as was assessed by 24-h sodium excretion (Table 1).

**Table 1:** Characteristics of the study subjects after LSD and HSD.

	HME patients (*n* = 7)		Healthy controls (*n* = 12)			
	LSD	HSD	p	LSD	HSD	p
**Body fluids**						
TBW (L)	46.3 (5.0)	47.7 (4.7)	<0.001	44.4 (4.0)	45.9 (4.0)	<0.01
ECFV (L)	14.1 (1.4)	14.3 (1.7)	0.68	15.1 (3.6)	17.1 (2.6)	0.06
PV (L)	3.2 (0.2)	3.3 (0.4)	0.39	3.4 (0.6)	3.6 (0.6)	0.23
IFV (L)	11.0 (1.4)	11.0 (1.6)	0.89	11.7 (3.3)	13.5 (2.3)	0.06
ICFV (L)	32.1 (4.7)	33.4 (4.8)	0.01	29.3 (4.0)	28.8 (4.2)	0.60
**Office BP**						
Supine systolic BP (mmHg)	118.4 (7.2)	119.1 (9.4)	0.80	117.3 (7.5)	118.8 (5.5)	0.33
Supine diastolic BP (mmHg)	61.1 (4.8)	63.9 (7.9)	0.08	58.3 (5.4)	57.4 (5.4)	0.28
Supine heart rate (Bpm)	63.4 (7.6)	60.9 (8.1)	0.36	56.0 (7.6)	56.9 (10.5)	0.48
**Plasma**						
NT-proBNP (ng/L)*	8.9 (8.1)	11.1 (21.1)	0.09	8.5 (6.7)	16.4 (27.9)	0.08
Sodium (mmol/L)	139.3 (1.9)	140.1 (0.9)	0.20	137.5 (1.6)	140.3 (1.8)	<0.01
Potassium (mmol/L)	3.8 (0.2)	3.9 (0.2)	0.16	3.9 (0.3)	3.9 (0.2)	0.76
Osmolality (mOsm/kg)	286.0 (2.2)	289.6 (2.4)	<0.01	284.9 (3.1)	289.6 (3.8)	<0.01
Effective osmolality(mOsm/kg)	269.3 (4.5)	272.1 (1.8)	0.18	267.9 (4.8)	273.6 (3.5)	<0.01
Bicarbonate (mmol/L)	26.9 (1.7)	25.1 (1.5)	0.13	25.9 (2.4)	25.4 (1.6)	0.88
Chloride (mmol/L)	98.0 (1.7)	101.6 (1.4)	0.001	99.6 (1.5)	103.3 (2.0)	<0.01
Urea (mmol/L)	4.3 (1.4)	4.2 (0.9)	0.82	4.9 (0.8)	4.9 (0.9)	0.74
**24** **h urine**						
Volume (mL/24 h)	1845.0 (573.0)	2008.3 (725.5)	0.61	1702.1 (550.7)	1908.9 (544.4)	0.25
Sodium (mmol/24 h)	23.8 (11.6)	291.9 (66.8)	<0.01	19.1 (9.5)	340.8 (104.1)	<0.01
Potassium (mmol/24 h)	69.5 (40.1)	87.3 (26.6)	0.07	87.7 (26.0)	89.5 (19.7)	0.93
Urea (mmol/24 h)	328.1 (185.1)	421.1 (146.0)	0.22	403.1 (106.3)	491.6 (88.2)	0.02
Chloride (mmol/24 h)	24. (8.2)	261.2 (60.4)	<0.01	23.3 (3.7)	340.8 (99.2)	<0.01
Osmolality(mOsm/kg)	314.4 (93.5)	653.0 (271.1)	<0.01	430.9 (164.4)	743.7 (164.8)	<0.01
Creatinine (mmol/24)	13.0 (3.4)	14.1 (2.7)	0.14	15.8 (2.4)	17.3 (2.5)	0.03

Data are depicted as mean (SD) or median (IQR). TBW, total body water. ECFV, extracellular fluid volume. PV, plasma volume. IFV, interstitial fluid volume. ICFV, intracellular fluid volume. BP, blood pressure. Bpm, beats per minute. NT-proBNP, N-terminal pro b-type natriuretic peptide. Data are tested using a paired t-test (LSD vs HSD) or Wilcoxon Rank test if marked with *.

**Fig. 1:**
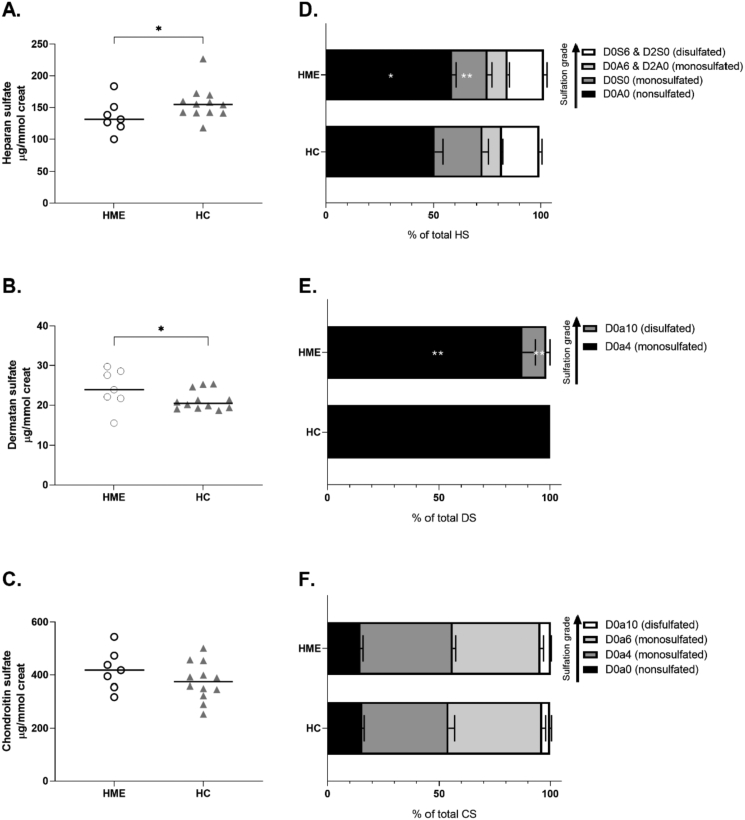
**Urinary 24-h GAG excretion at LSD. A.** During LSD, HME patients had significantly lower HS excretion in 24-h urine samples when compared to HC (131.3 (31.0) μg/mmol creatinine versus 154.7 (25.5), *p* = 0.03). **B.** During LSD, dermatan sulfate excretion tended to be slightly higher in HME patients (23.9 (6.9) μg/mmol creatinine vs 20.5 (4.6) μg/mmol creatinine, *p* = 0.050).**C.** No differences in chondroitin sulfate excretion could be observed (374.0 (111.4) μg/mmol creatinine vs 417.8 (118.1) μg/mmol creatinine, *p* = 0.43). **D.** There were significantly more nonsulfated disaccharides (D0A0) of heparan sulfate in HME patients than in healthy controls. The notation is as follows [32]: D0A0 = ΔUA-GlcNAc, D0S0 = ΔUA-GlcNS, D0A6 = ΔUA-GlcNAc6S, D2A0 = ΔUA2S-GlcNAc, D0S6 = ΔUA-GlcNS6S, D2S0 = ΔUA2S-GlcNS. **E.** In healthy controls, all the dermatan sulfate consisted of D0a4 disaccharides, while in HME patients also D0a10 disaccharides were measured. The notation is as follows [32]: D0a4 = ΔUA-GalNAc4S, D0a10 = ΔUA-GalNAc4S6S. **F.** The percentages disaccharides of chondroitin sulfate were equal among HME and healthy controls. The notation is as follows [32]: D0a0 = ΔUA-GalNAc, D0a4 = ΔUA-GalNAc4S, D0a6 = ΔUAGalNAc6S, D0a10 = ΔUA-GalNAc4S6S. HC, healthy controls. HME, hereditary multiple exostoses. HS, heparan sulfate. DS, dermatan sulfate. CS, chondroitin sulfate. *P ≤ 0.05 vs. healthy controls. **P < 0.01 vs. healthy controls. Data are presented as individual data with median and IQR. Data were tested with a Kolmogorov-Smirnov test.

During LSD, median HS excretion in 24-h urine samples was 15.3% lower in HME patients when compared to healthy controls, whilst, median dermatan sulfate excretion was borderline significantly higher in HME patients with 14.3% (Fig. 1A, B). Of their total HS excretion, the amount of less-sulfated HS (D0A0) was higher in HME patients (Fig. 1C).

### 3.2 Distinct body fluid distribution shifts in HME patients in responses to HSD

**Fig. 2:**
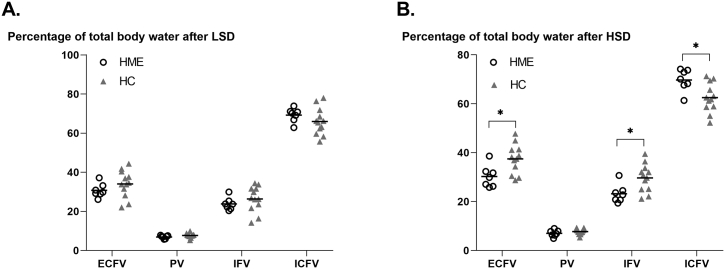
**Distribution of body fluid compartments over TBW. A.** After LSD, body fluid compartments are similar divided over TBW **B.** After HSD, the ECFV compartment of TBW is larger in HC, which mainly results form a larger IFV compartment. In HME patients, the ICFV compartment is larger when compared with healthy controls. HSD, high sodium diet. LSD, low sodium diet. HME, hereditary multiple exostoses. HC, healthy controls. ECFV, extracellular fluid compartment. PV, plasma volume. IFV, interstitial fluid compartment. ICFV, intracellular fluid compartment. Individual data points are presented with their group mean. Data were tested with an independent *t*-test. *p ≤0.05.

During LSD, TBW as similarly distributed among the different body fluid compartments in both HME and healthy controls (Fig. 2A). High sodium intake resulted in a different distribution of TBW across body fluid compartment between groups (Fig. 2B), while absolute TBW increase was not different between HME and healthy controls (1.4 L vs 1.5 L, *p* = 0.91). After HSD, relative proportions of ECFV and IFV to TBW became significantly lower in HME as compared to healthy controls, whereas the proportion of ICFV to TBW became significantly higher in HME (Fig. 2B). In HME patients, the ICFV rise amounted 3.9 ± 3.0% (*p* = 0.01) as compared to LSD, but did not significantly change in healthy controls (−1.4 ± 10.6%, *p* = 0.66). A similar pattern for absolute ICFV and ECFV rise was observed in both groups, with ICFV expansion in HME patients (1.2 L vs −0.5 L) and mainly ECFV expansion in healthy controls (0.2 L vs 2.0 L) (Table 1). In both groups, no HSD-induced changes in BP were observed (Table 1).

### 3.3 Altered osmoregulation in response to HSD in HME patients

**Fig. 3:**
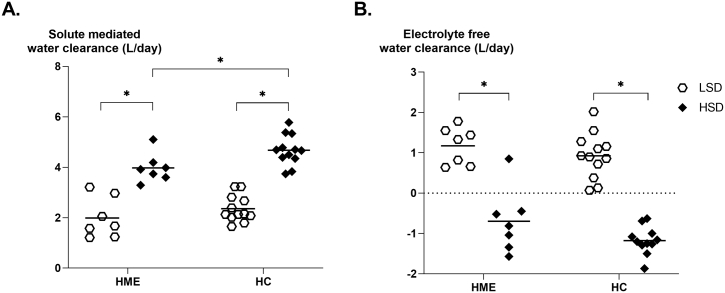
**Urine clearance during HSD and LSD. A.** Both groups significantly increase their solute mediated water clearance (L/day). However, during HSD, HC have more solute mediated water clearance than HME patients (4.0 vs 4.7 L/day, *p* = 0.02). **B.** Both groups significantly decrease their electrolyte free water clearance. During HSD, electrolyte free water clearance was not significantly different between HME and HC, respectively −0.7 vs −1.2 L/day, *p* = 0.09. HSD, high salt diet. LSD, low salt diet. HME, hereditary multiple exostoses. HC, healthy controls. Individual data points are presented with their group mean. Data were tested with an paired *t*-test or independent t-test (HME vs HC). *p ≤0.05.

Besides IFV expansion, we also observed an increase of plasma sodium and calculated effective osmolality after HSD in healthy controls, while in HME patients plasma sodium and calculated effective osmolality did not change (Table 1). Plasma chloride and plasma osmolality increased equally in both groups. Looking at urinary clearances after HSD, electrolyte free water clearance became more negative in both groups, whereas the solute mediated, but not electrolyte mediated, water clearance was significantly lower in HME as compared to healthy controls (Fig. 3).

### 3.4 Changes in total HS correlate With fluid volume in healthy controls

**Fig. 4:**
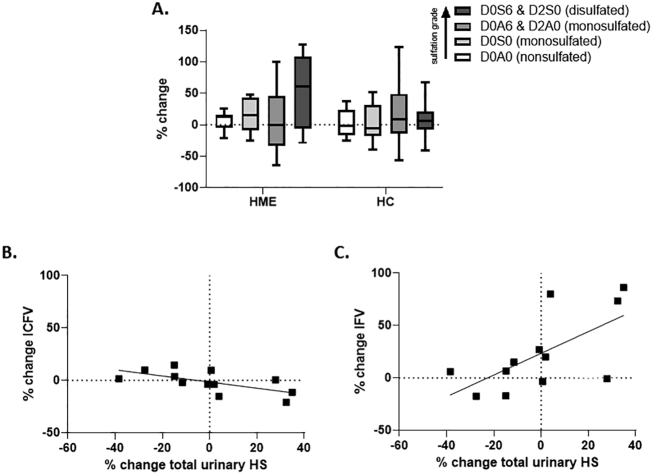
**Change in urinary HS concentration (**μ**g/mmol creat) correlates with changes in ICFV and IFV. A.** Both groups show a highly heterogeneous percentage change in sulfated and nonsulfated disaccharides of HS. The notation is as follows [25]: D0A0 = ΔUA-GlcNAc, D0S0 = ΔUA-GlcNS, D0A6 = ΔUA-GlcNAc6S, D2A0 = ΔUA2S-GlcNAc, D0S6 = ΔUA-GlcNS6S, D2S0 = ΔUA2S-GlcNS. **B.** Linear regression graph showing the correlation between percentage change in total urinary HS and percentage change of ICFV in healthy controls, showed a significant negative correlation (*r* = −0.76, *p* < 0.01). **C.** Linear regression graph showing a significant positive correlation between percentage change of total urinary HS and percentage change of IFV (*r* = 0.68, *p* = 0.02) in healthy controls. HC, healthy controls. HS Heparan sulfate. ICFV, intracellular fluid volume. IFV, interstial fluid volume. Individual data points are presented with their group mean. Correlation was tested with a Spearman rank test (B,C).

In both groups, HSD induced a highly heterogeneous percentage change (relative to LSD) in total urinary HS and HS disaccharides (Fig. 4A). While in healthy controls, urinary HS had a significant negative correlation with the change in ICFV (Fig. 4B), the correlation between urinary HS and ICFV was absent in HME patients (*r* = 0.11, *p* = 0.82). The percentage change in total urinary HS also showed a positive correlation with percentage change of IFV, while also this correlation was absent in HME patients (Fig. 4C).

## 4 Discussion

The aim of this study was to assess if HME patients, who are characterized by defective HS polymerization, respond differently to high and low dietary sodium exposure with regard to their sodium, osmotic and water balance. At low sodium conditions, no signs of disturbed body fluid volume regulation nor osmoregulation could be observed. After switching to a hypertonic condition by excessive sodium consumption, the division of water over the body fluid compartments markedly changed. In HME patients, HSD resulted in a larger ICFV without concurrent changes in plasma effective osmolality, while in healthy controls, ECFV increased (predominantly as a result of IFV expansion) as well as effective plasma osmolality. In addition, we found a highly significant negative association between sodium diet-induced changes in total urinary HS excretion and ICFV change in healthy controls. Together, these findings might implicate that intact HS polymerization is important for regulation of intracellular water during high sodium conditions.

Our findings show disturbed osmoregulation regulation in response to HSD leading to ICFV expansion in HME patients. Considering the impact of disturbed osmoregulation that reflects intracellular fluid excess on mortality and morbidity in various other populations, our findings may have significant impact on long-term health outcomes of HME patients [[33], [34], [35]]. Also, our results show that HS composition may play a crucial role in sodium homeostasis. The widely accepted concept of internal environment maintenance is based on the perception of iso-osmolality between the extra- and intracellular space [36]. According to this view changes in intracellular or extracellular solute content result in the immediate flow of water into or out of the cell, until equilibrium is achieved again. In HME patients, the hypertonic condition induced, however, a distinct fluid distribution in which a water shift - in order to prevent HSD-induced increases of plasma sodium and effective osmolality - generated ICFV expansion. Whilst, in healthy controls we observed an increase in IFV, as well as in plasma sodium and effective osmolality but maintenance of ICFV. Our results suggests that HS polymerization may be involved in the maintenance of intracellular osmolality, and that perturbed HS polymerization, as observed in HME patients, leads to increased ICFV and potentially detrimental cellular water shifts.

In line with the observed shift in intra- and extracellular water distribution in HME patients, also the urinary handling of water, sodium and other solutes indicates more water retention in this group. No differences in 24-h urinary volume could be observed in both groups. However, healthy controls, as compared to HME patients, showed higher solute mediated water excretion in response to HSD. In both groups, we showed that electrolyte free water clearance was negative – an effect that, in the light of maintaining the internal environment, can be interpreted as an appropriate response of water retention during hypertonic conditions. Yet, in response to expansion of the intracellular space, as we did observe in HME patients, increased water excretion by the kidneys would have been expected in these patients. Regarding body fluid regulation, recent animal and human studies have shown that conclusions cannot be drawn without exploration of both sodium as well as urea regulation [[37], [38]]. It has been shown in human and mice that the renal response to high dietary salt conditions relies on the maintenance of negative free water clearance and increased renal medullary urea accumulation, in order to safe water and excrete excess sodium [[37], [38]]. Indications of renal urea preservation could not be observed in our study, yet the healthy controls showed increased urea excretion after HSD, possibly explaining increased solute mediated water clearance in this group. Since HS is the most abundant GAG expressed in renal tissue [39], impaired body fluid regulation in HME patients suggests that, besides the recently uncovered natriuretic-ureotelic regulation, intact HS biosynthesis plays an important role in body fluid volume homeostasis and should be considered in further research. In line with this hypothesis, an animal study showed increased sulfation of renal HS in healthy rats fed a high sodium diet, which was associated with renal tissue remodeling events [40]. Interestingly, HS sulfation enzymes upregulated by sodium in these experiments modify HS chains after EXT1/EXT2 HS polymerization, which suggests renal HS response to HSD might be different in HME patients.

The importance of HS polymerization in the maintenance of plasma osmolality and water distribution is further exemplified by the finding that in healthy controls ICFV showed a strong negative association with percentage delta change of total urinary HS. This might indicate that an adequate response of extracellular HS increase – allowing increased interstitial tissue sodium accumulation– is necessary to protect ICFV increase. This is in agreement with our previous study, in which we showed that in response to HSD healthy controls showed dermal HS modification, whilst HS modification was absent in HME patients [27]. Besides the skin also the endothelial surface layer, a thin dynamic HS-rich layer covering endothelial cells, has been identified as an important compartment for sodium accumulation and sodium balance [[41], [42], [43]]. In mice with combined heterozygous loss of EXT1 and EXT2, it was found that that endothelial surface layer thickness was reduced up to 77% [[15], [44]]. Taken together, our data supports our hypothesis that intact HS modification during HSD is important for sodium and water balance.

To our knowledge this is the first study investigating the influence of HSD on sodium homeostasis and the water balance in HME patients, characterized by altered HS polymerization. We conducted a controlled randomized dietary sodium loading intervention study in matched groups and precisely measured ECFV and PV. On day eight of our intervention a new steady state in sodium and water balance is expected to be achieved [45]. Furthermore, previous studies showed significant tissue remodeling and local osmoregulatory responses after one week of HSD [[27], [46]]. However, certain limitations need to be considered. Firstly, for this physiological study we only included young male participants, which are not representative for the entire HME patient population. Since responses to sodium are age- and sex-related, further research is necessary in older and female HME patients. Yet, the observation that body fluid volume regulation and osmolality are already affected at young age underlines the importance of studying osmoregulation in this patient group. Secondly, we were unable to measure tissue sodium content and tissue GAG composition directly. As a consequence, we had to rely on 24-h urinary GAG excretion. In low sodium conditions, HME patients showed changed urinary GAG composition, but it is unknown how this reflects the GAG content in plasma and tissue. Furthermore, our sample size is, due to the prevalence of HME, small. Considering the increased risk for type II errors (false negative findings), we performed a post-hoc power calculation for our primary end points (body fluid volumes). This analysis nevertheless showed that no other outcome was to be expected if we would have been able to increase our sample size to 200 HME participants.

## 5 Conclusions

HME patients, characterized by defective HS polymerization, show altered body fluid distribution and osmoregulatory responses after HSD, with indications for reduced extracellular sodium accumulation capacity in the interstitium and reduced capacity in maintaining a stable the milieu interieur. As a consequence, intracellular fluid handling changes after hypertonic stress in HME patients. Whether these sequelae may impact health outcomes of HME patients on the long term remains to be confirmed. Notwithstanding, our findings provide additional support that HS is crucial for sodium homeostasis and the body fluid balance.

## Authors' contributions

LV conceptualized the clinical trials. ROE and NR conducted the investigation. NV performed measurements of urinary glycosaminoglycans. HV supervised the radiolabeled albumin-based plasma volume measurements. JO and YC analyzed the data. JO wrote the manuscript. All authors reviewed the manuscript. LV and BJvdB supervised the entire process. All authors read and approved the final manuscript.

## Funding

This work was supported by the 10.13039/501100002997Dutch Kidney Foundation (Junior Kolff grant number KJPB 11.22 and Senior Kollf grant number 18OKG12 to LV) and The Netherlands Organization for Health Research and Development (clinical fellowship grant number 90700310 to LV).

## Ethics approval and consent to participate

The Academic Medical Center ethics committee approved the conductance of the studies at the Amsterdam UMC, location AMC (Amsterdam, the Netherlands). All subjects provided written informed consent before the start of the study. The trials were conducted in accordance with the Declaration of Helsinki and according to the original protocol, which was registered in the Netherlands trial register (www.trialregister.nl; NTR4095, NTR4788).

## Declaration of Competing Interest

All authors have read the journals policy on disclosure of potential conflicts of interest. There are no conflicts of interest to declare.
