# Supplementary material for: Perturbed body fluid distribution and osmoregulation in response to high salt intake in patients with hereditary multiple exostoses
Source: Mol Genet Metab Rep. 2021 Nov 5;29:100797. doi: 10.1016/j.ymgmr.2021.100797 (PMC8591465; doi:10.1016/j.ymgmr.2021.100797)
Supplement: Supplementary material [file mmc1.docx]

**SUPPLEMENT**

**Supplementary table 1. Baseline characteristics of study subjects**

|  | **HME patients**  **(n=7)** | **Healthy controls (n=12)** | **p-value** |
| --- | --- | --- | --- |
| **General characteristics** |  |  |  |
| Age (year) | 26.6 (8.5) | 22.7 (4.1) | 0.29 |
| Total body weight (kg) | 78.8 (7.4) | 75.7 (6.8) | 0.37 |
|  |  |  |  |
| **Office BP** |  |  |  |
| Supine systolic BP (mmHg) | 117.9 (4.3) | 121.3 (7.8) | 0.31 |
| Supine diastolic BP (mmHg) | 63.2 (6.3) | 59.0 (6.5) | 0.21 |
| Supine heart rate (bpm) | 63.6 (9.3) | 61.0 (7.0) | 0.54 |
|  |  |  |  |
| **Plasma** |  |  |  |
| Hemoglobin (mmol/L) | 9.3 (0.3) | 9.3 (0.5) | 0.89 |
| Sodium (mmol/L) | 140.1 (0.9) | 140.0 (1.8) | 0.85 |
| Potassium (mmol/L) | 4.0 (0.3) | 4.2 (0.2) | 0.16 |
| Osmolality (mOsm/kg) | 289.7 (3.3) | 286.6 (16.4) | 0.63 |
| Urea (mmol/L) | 4.3 (0.8) | 5.4 (1.1) | 0.03 |
| Creatinine (umol/L)* | 74.0 (8.0) | 79.0 (18.5) | 0.12 |
| eGFR (ml/min/1.73m^2^) | 121.6 (6.3) | 114.7 (12.1) | 0.18 |
|  |  |  |  |
| **24h urine** |  |  |  |
| Volume (ml/24h) | 2014.7 (870.0) | 1623.8 (558.5) | 0.26 |
| Sodium (mmol/24h) | 153.7 (62.2) | 166.0 (70.6) | 0.71 |
| Potassium (mmol/24h) | 71.9 (10.0) | 71.3 (16.1) | 0.93 |
| Urea (mmol/24h) | 411.6 (147.9) | 485.0 (202.6) | 0.50 |
| Osmolality (mOsm/kg) | 515.3 (227.9) | 663.7 (227.3) | 0.19 |
| Creatinine (mmol/24h) | 14.7 (2.1) | 16.4 (2.7) | 0.17 |

*Data are depicted as mean (SD) or median (IQR). Data was obtained at screening before commencement of the diets. BMI, body mass index. BP, blood pressure. Bpm, beats per minute. eGFR, estimated glomerular filtration rate based on CKD-EPI. Data are tested using an independent t-test or Kolmogorov-Smirnov test for non-normal distributed variables marked with *.*
